# Monitoring Surface Deformations in a Fossil Landslide Zone and Identifying Potential Failure Mechanisms: A Case Study of Gümüşhane State Hospital

**DOI:** 10.3390/s24154995

**Published:** 2024-08-01

**Authors:** Selçuk Alemdag, Sefa Yalvaç, Olga Bjelotomić Oršulić, Osman Kara, Halil İbrahim Zeybek, Hasan Tahsin Bostanci, Danko Markovinović

**Affiliations:** 1 Department of Geology Engineering, Gumushane University, 29100 Gumushane, Turkey; 2 Department of Geomatics Engineering, Gumushane University, 29100 Gumushane, Turkey; sefayalvac@gumushane.edu.tr (S.Y.); htbostanci@gumushane.edu.tr (H.T.B.); 3 Department of Geodesy and Geomatics, University North, 42000 Varazdin, Croatia; oborsulic@unin.hr (O.B.O.);; 4 Department of Civil Engineering, Gumushane University, 29100 Gumushane, Turkey; 5 Department of Geography, Ondokuz Mayıs University, 55270 Samsun, Turkey

**Keywords:** landslide, LE and FEM-SSR methods, inclinometer, geodetic monitoring, GNSS

## Abstract

The escalating occurrence of landslides has drawn increasing attention from the scientific community, primarily driven by a combination of natural phenomena such as unpredictable seismic events, intensified precipitation, and rapid snowmelt attributable to climate fluctuations, compounded by inadequacies in engineering practices during site selection. Within the scope of this investigation, contemporary geodetic techniques using the GNSS were employed to monitor structural and surface deformations in and around a hospital edifice situated within an ancient fossil landslide region. Additionally, inclinometer measurements facilitated the determination of slip circle parameters. A subsequent analysis integrated these datasets to scrutinize both the hospital structure and its surrounding slopes. In addition to the finite element method, four different limit equilibrium methods (Bishop, GLE–Morgenstern–Price, Spencer, and Janbu) were used in the evaluation of stability. Since the safety number determined in all analyses was <1, it was determined that the slope containing the hospital building was unstable. The movement has occurred again due to the additional load created by the hospital building built on the currently stable slope, the effect of surface and groundwater, and the improperly designed road route. As a result of geodetic monitoring, it was determined that the sliding speed on the surface was in the N-E direction and was approximately 3 cm, and this situation almost coincided with inclinometer measurements.

## 1. Introduction

Mass movements represent significant natural disasters that exert adverse impacts on both the environment and human livelihoods. Fossil landslide regions have the potential for reactivation, triggered by seismic activity, intense and abrupt precipitation, engineering operations like excavation and blasting, and the imposition of additional loads on slopes from structures such as shelters, schools, and hospitals [1]. Beyond inflicting economic and environmental repercussions, landslides detrimentally impinge on human well-being, compounded by their unpredictable nature, thereby heightening awareness and concern surrounding the issue over time.

Many studies have been conducted on landslide investigations from past to present, and these studies have been mostly evaluated using limit equilibrium or numerical analysis methods [2,3,4,5,6,7,8,9,10,11,12,13,14]. However, both of these methods do not reveal important parameters such as the sliding depth of a mass movement and the possible sliding velocities. In efforts to monitor landslide areas both regionally and locally and to preemptively address potential risks, on-site methods including inclinometer measurements and electrical resistivity tomography enable the determination of parameters such as sliding depth, velocity, and superficial deformations of the moving mass [15,16].

In addition to the aforementioned methodologies, surface deformations can also be monitored spatially and at specific points using geodetic techniques. Thus, mass movements can be estimated in 3D with the GNSS method, and the change in deformation with depth can be clearly revealed with the inclinometer measurements. The Global Navigation Satellite System (GNSS), adopting a point-based approach, offers a robust and precise method for monitoring changes in the Earth’s surface at designated locations over time. By installing GNSS receivers in the expected deformation area, researchers can continuously gather data on the positions of these points relative to a global reference frame established by satellites within the GNSS constellation [17,18]. This technique boasts high accuracy, is capable of detecting even subtle movements with sub-centimeter precision, and offers continuous monitoring capabilities, facilitating data collection over prolonged durations to discern deformation behaviors [19,20]. Furthermore, the GNSS allows for both horizontal and vertical displacement, thereby providing comprehensive insights into surface deformation processes [21,22]. Thanks to its advantages such as low cost, the ability to measure day and night, and high precision in determining deformations in 3D with the accuracy of a few millimeters, it has been one of the most frequently used methods for determining surface deformations for many years [23,24].

**Figure 1:**
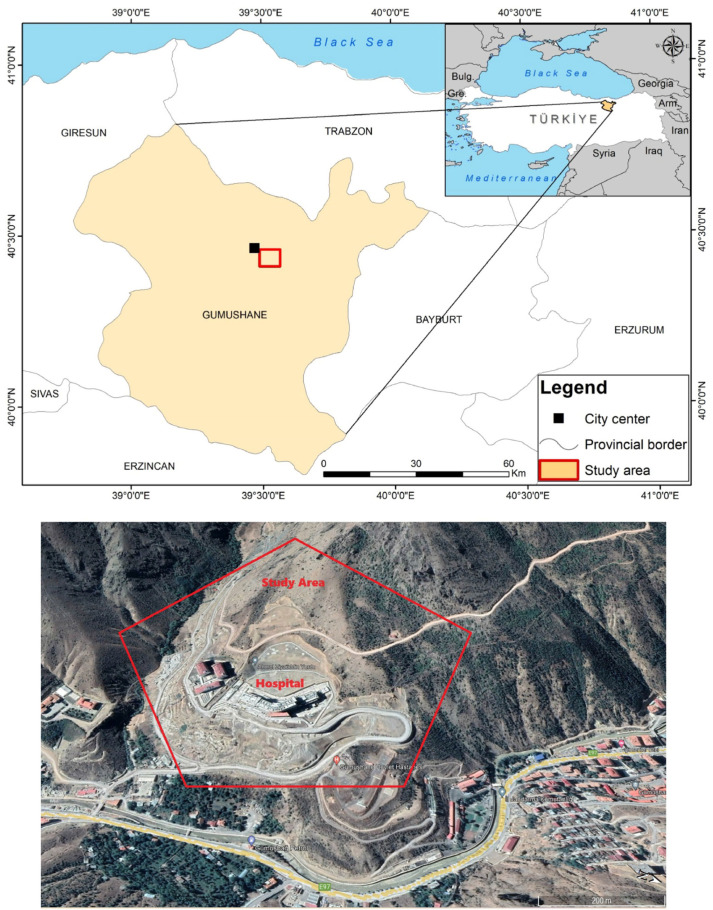
Map illustrating the study area’s location and an image of the location site from Google Earth.

In this study, the causes of surface deformations occurring in and around Gümüşhane State Hospital, built in the Eskibağlar District (Gümüşhane) of the Şehbenderler locality (Figure 1), which is an old fossil landslide area, were investigated, and the reasons for the cracks occurring in the columns, beams, and walls of the hospital and the deformations in the surrounding reinforced concrete structures were revealed. Both geodetic and geotechnical investigations were carried out to determine the causes. Since the State Hospital is actively used, determining the failure mechanism of possible mass movement is of vital importance in terms of loss of life and property. Furthermore, employing integrated methodologies to ascertain the lithological complexity of fossil landslide areas and determine the depth and origin of movement will provide insights into resolving the issue. Among these techniques, conducting inclinometer measurements within boreholes in the field to delineate the depth of the slip circle, and monitoring vertical and horizontal deformations in the hospital building (by using GNSS), all contribute to tackling such intricate engineering challenges. It is imperative to underscore that, hitherto, landslide studies have not integrated analyses to assess the speed, direction, and depth of movement in both regional and local contexts. Hence, emphasizing the importance of monitoring both regional and local movements in evaluating the reactivation of fossil landslide areas is paramount. Deformations obtained using monitoring methods, building load, and slope stability under the influence of maximum horizontal ground acceleration that will occur in a possible earthquake due to the region’s proximity to the North Anatolian Fault (approximately 90 km) were evaluated by both the limit equilibrium analysis and numerical analysis method, taking into account possible risks. It has been determined whether the hospital will be used or not. Thus, this study aimed to reveal the mass movement in the study area in three dimensions, to verify the deformations with different monitoring methods, so as to provide the most comprehensive information to different disciplines for landslide prevention studies.

## 2. Geological Setting of the Study Area

**Figure 2:**
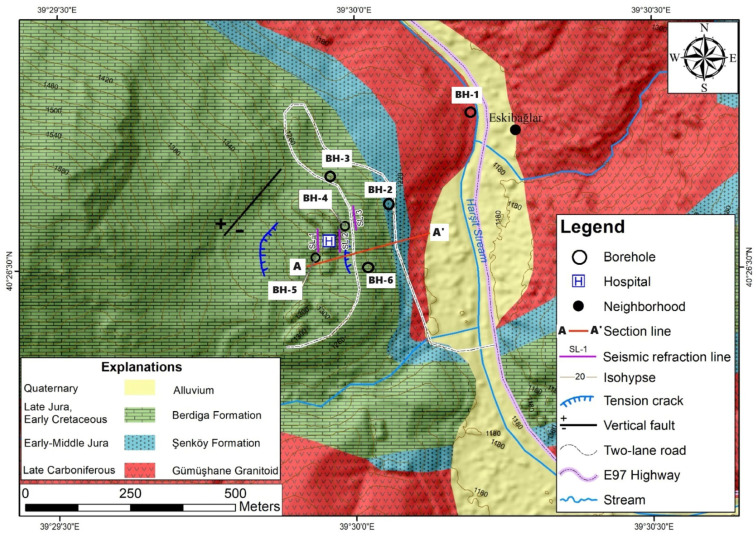
Engineering geology map of the study area Within the study area, the precise topographic slope of the fossil landslide region was delineated utilizing digital elevation model (DEM) data, characterized by a resolution of 5 m. Notably, the hospital premises and its environs exhibit slopes ranging from 20° to 60°. Upon scrutiny of the slope map (Figure 5), it becomes evident that the hospital’s construction site comprises limestone blocks that have descended due to normal faulting.

The geological investigations conducted in the study area revealed the presence of several distinct geological units. The Late Carboniferous-aged Gümüşhane Granitoid, the Early-Middle Jurassic-aged Şenköy Formation, and the Late Jurassic-Early Cretaceous-aged Berdiga Formation are prominently exposed (Figure 2).

**Figure 3:**
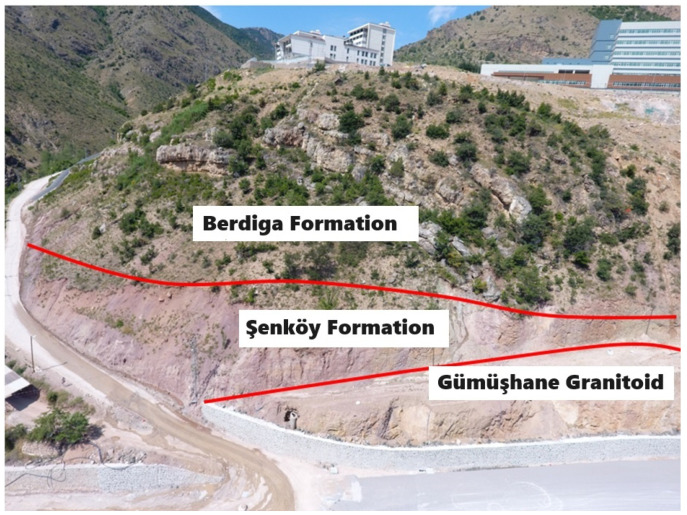
View of the formations outcropping in and around the study area.

Upon closer examination, the Late Carboniferous-aged Gümüşhane Granitoid predominantly comprises granodiorite and granite derived from the continental crust, with sporadic occurrences of quartz diorite, quartz monzonite, dacite, and rhyolites [25,26]. Aplitic dykes are occasionally observed within the granitic complexes. Located at the lowermost section of the study area, the Gümüşhane Granitoid exhibits extensive decomposition in certain areas, forming arenas, and is characterized by well-defined joint sets (Figure 3).

The Early-Middle Jurassic-aged Şenköy Formation overlies the Late Paleozoic basement rocks unconformably. It commences with a basal conglomerate composed of quartzite and granite pebbles, succeeded by coal-bearing interlayered sandstones overlain by red limestones (Ammonitico Rosso facies) rich in macrofossils [27]. This formation further comprises a sequence of conglomerate, sandstone, siltstone, and marl layers, occasionally interspersed with basic volcanic materials such as tuff, tuffite, andesite, spilitic basalt, lava, and pyroclastics. Compared to other units in the study area, the Şenköy Formation is thinly distributed, with claystone–marl layers separated and dispersed as clay in the field (Figure 3).

The Late Jurassic-Early Cretaceous-aged Berdiga Formation, a significant stratigraphic marker in the Eastern Pontides, unconformably overlays the Early-Middle Jurassic Şenköy Formation. This formation comprises medium-thick layered to occasionally massive dolomitic limestones at its base [28,29]. Conglomerate, sandstone, and siltstone successions dominate the upper levels, terminating in limestone, dolomite, and dolomitic limestone layers.

**Figure 4:**
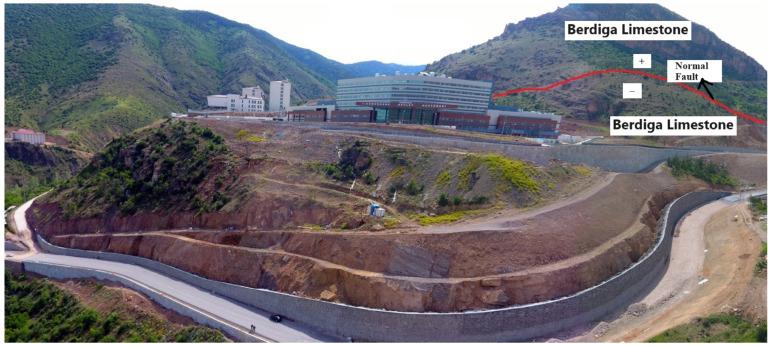
Faulting in the limestones of the Berdiga Formation in the study area.

The foundation of the Gümüşhane State Hospital building, situated in the study area, rests upon the limestones of the Berdiga Formation, which are displaced by a normal fault system (Figure 4). Additionally, the aquifer properties of the Berdiga Formation’s limestones facilitate the discharge of surface flow rainwater from the impermeable claystone–marl and clay layers of the Şenköy Formation at its base, preventing water accumulation along cracks and layer planes.

**Figure 5:**
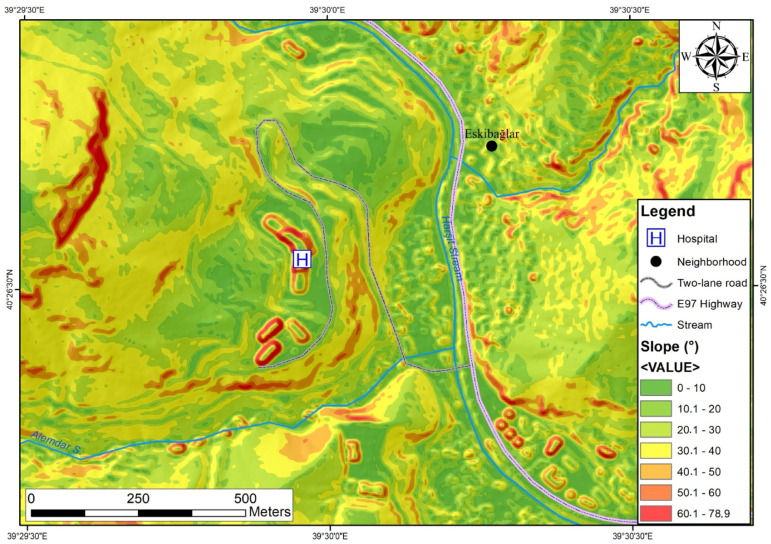
Topographic slope map depicting the study area’s terrain characteristics.

## 3. Engineering Investigation in the Study Area

The Gümüşhane State Hospital occupies an area atop Berdiga limestone formations that have been affected by a normal fault system, thereby positioning it within a fossil landslide zone. Reengineering structures within such geologically challenging areas presents considerable difficulty, necessitating meticulous engineering investigations.

**Figure 6:**
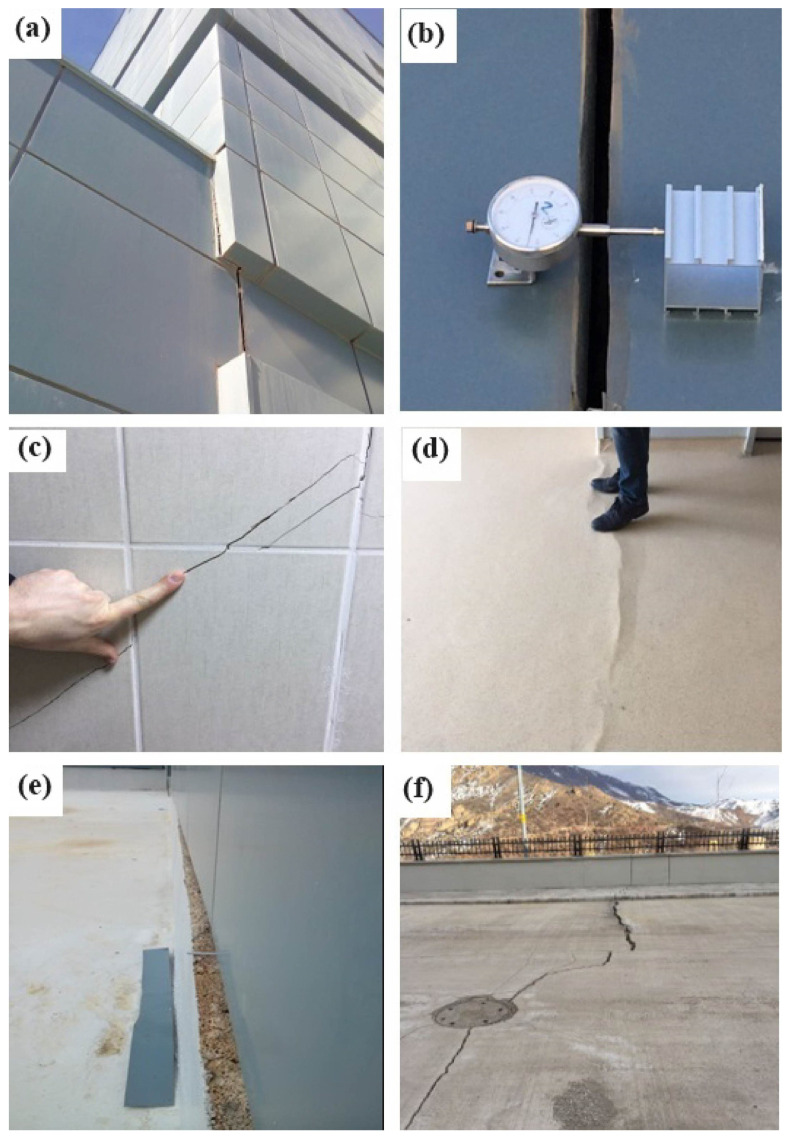
(**a**,**b**,**e**) Opening and torsion in the dilation gap between hospital blocks; (**c**) cracking in the moldings inside the building; (**d**) deformation in the corridor inside the hospital; and (**f**) collapses in the hospital garden.

**Figure 7:**
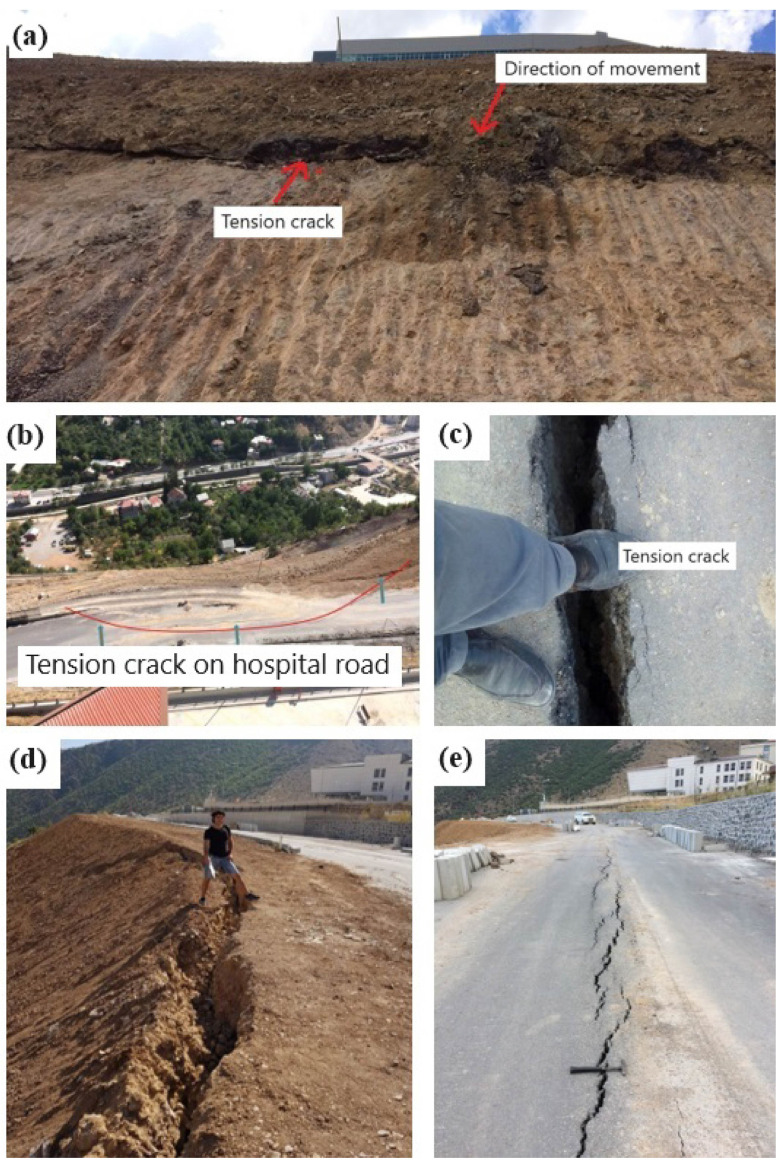
(**a**) Stress cracks formed on the slope under the hospital; (**b**,**c**,**e**) stress cracks formed on the hospital road; and (**d**) stress cracks formed in the landscape area.

Due to insufficient engineering inquiries in the study vicinity, deformations have manifested both within the constructed and actively utilized State Hospital (Figure 6), and the reinforced concrete walls within landscaped parks and garden areas (Figure 7). The GNSS method, one of the most well-known geodetic monitoring techniques, has been employed to monitor the movement mechanism affecting the hospital building, adjacent roads, and landscaped areas within the study zone, and to identify potential mass movements. Furthermore, research drillings have been conducted as part of inclinometer measurements to delineate lithological transitions and potential slip circles, with subsequent laboratory experiments conducted on extracted samples.

In addition to these investigations, geophysical methods such as seismic refraction and Multichannel Analysis of Surface Waves (MASW) methods have been undertaken along three designated lines (SL1-2-3) surrounding the hospital. These endeavors aimed to ascertain alterations in geological formations with depth and determine the dynamic elastic parameters of the rock masses. The amassed data from field surveys and laboratory analyses will be utilized to assess slope failure mechanisms through limit equilibrium analysis and numerical analysis methodologies.

### 3.1. Inclinometer Measurements

In order to ascertain the underlying causes behind the emergence of cracks within the State Hospital premises in the study area and the superficial deformations evident in its vicinity, inclinometer measurements were conducted through the drilling of boreholes. Specifically, a borehole labeled as BH-4, drilled to a depth of 60 m, was positioned in front of the hospital building, while another borehole, BH-5, was drilled to a depth of 70 m at the rear (Figure 2).

**Figure 8:**
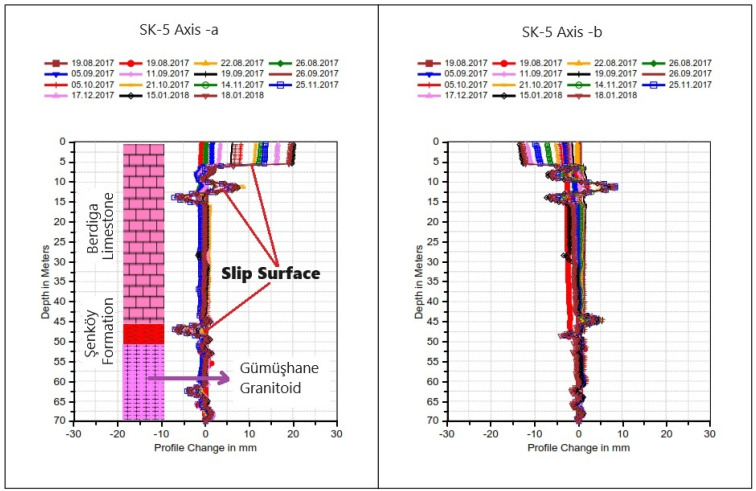
Cumulative inclinometer graph of well No. BH-5 located behind the hospital.

Among these boreholes, BH-5, situated behind the hospital, exhibited notable deformations. Following the initial reading on 19 August 2017, and subsequent readings over 14 periods culminating in the last observation on 18 January 2018, deformations were documented. At the fifth meter, approximately 20 mm of deformation was recorded, diminishing to approximately 12 mm at the eleventh meter and further reducing to 8 mm at the forty-fifth meter (refer to Figure 8). Notably, fluctuations in deformation observed in this well at the depth of 45 m and beyond consistently converged at nearly identical points during each reading period; these fluctuations are indicative of depth-dependent deformations within the borehole.

**Figure 9:**
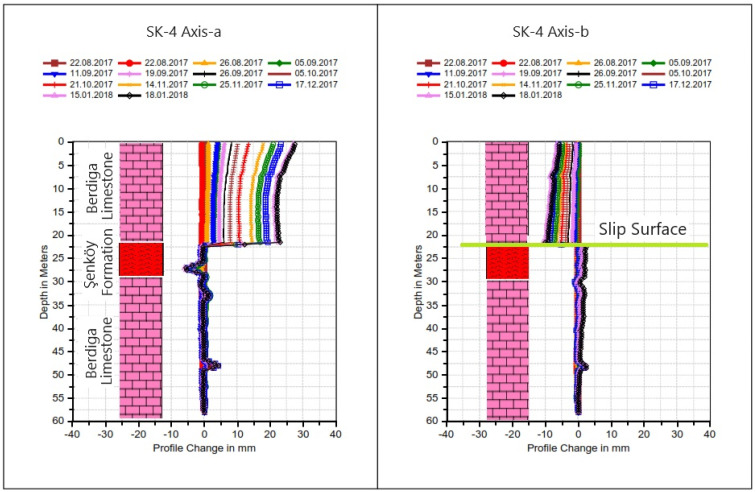
Cumulative inclinometer graph of well No. BH-4 located in front of the hospital.

The well No. BH-4 is in the front garden of the hospital, and at the end of 13 periods of reading after the first reading (22 August 2017) (last reading 18 January 2018), a deformation of approximately 25 mm was measured in the well at the 21st meter (Figure 9). The deformation fluctuations observed at the 27th and 48th meters in this well are due to depth-dependent deformations in the well.

Considering the deformations in the inclinometer measurements made in the boreholes SK5 and SK4 in the study area, the hospital building built in the fossil landslide area remained in the moving mass area, and the fossil landslide area became active again.

### 3.2. Geodetic Monitoring

In this section, the GNSS analyses that were performed to estimate the surface deformations occurring in the Gümüşhane State Hospital structure and its immediate surroundings are discussed.

#### Geodetic Network Design and GNSS Analysis

During on-site inspections conducted in 2017, it was observed that the hospital building comprises three distinct independent units: the central main section with eight floors, and two adjacent subdivisions, each with five floors, situated to the north and south of the main structure (see Figure 1). Given the differential deformation behaviors exhibited by these distinct sections, a total of five GNSS stations were installed on the roof of the main section (named as NKT1, NKT2, NKT3, NKT4, and NKT5), while an additional two stations were positioned on the roofs of the buildings in the northern and southern sections (labeled as NKT6 and NKT7, respectively).

Furthermore, four GNSS sites were established in close proximity to the hospital building. Two of these sites are situated in the eastern section (identified as KORK and KAYA), while the remaining two are positioned in the northern and southern sections of the building (referred to as ARKA and ISTI, respectively). Lastly, the GUMU CORSs (Continuously Operating Reference Stations), located 7 km away from the deformation area, were incorporated into the analyses to serve as a fixed reference point for estimating deformations.

**Figure 10:**
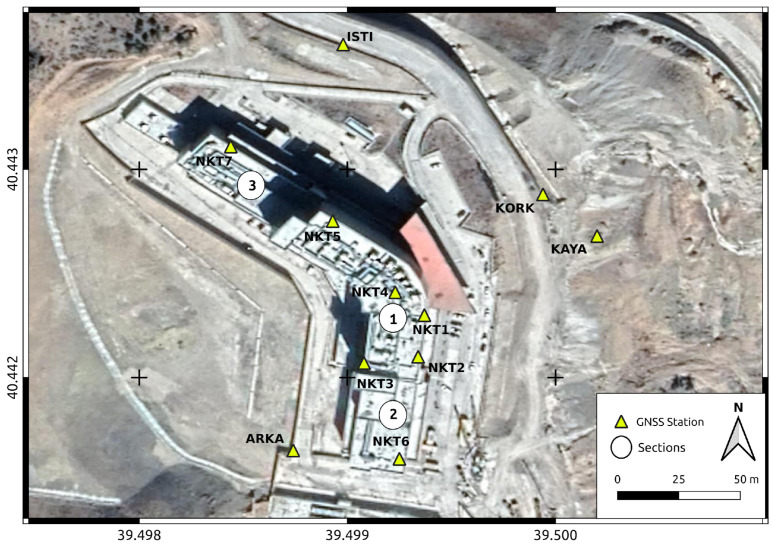
Deformation monitoring area and established GNSS stations.

Consequently, a comprehensive GNSS monitoring network comprising a total of 12 points was designed. The spatial arrangement of GNSS sites within the deformation monitoring area is depicted in Figure 10.

**Figure 11:**
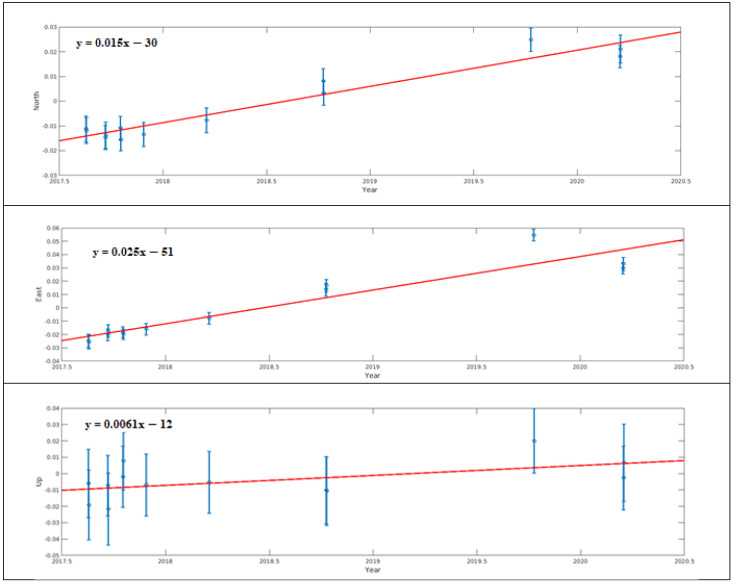
Time series with linear trend (red line) and slope values (blue line) created for north, east, and upward directions at NKT1 GNSS site (in meters).

**Figure 12:**
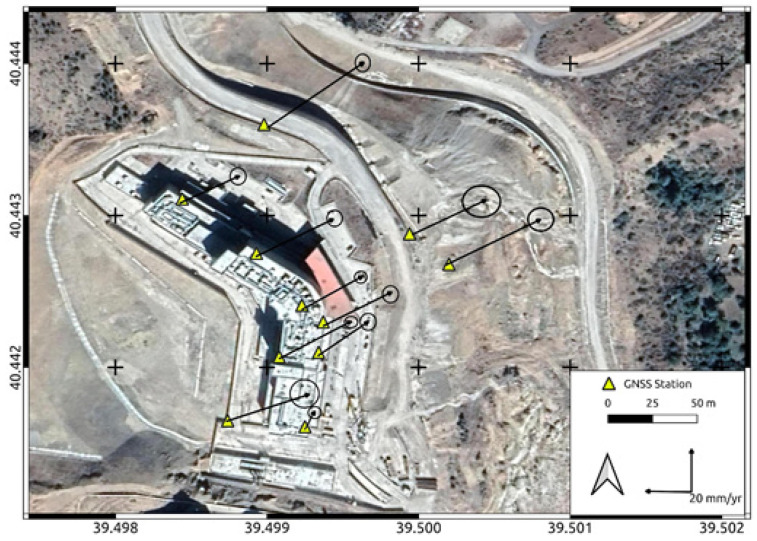
Deformation vectors derived from Kalman filter analysis with the error ellipses.

A total of eight periods of GNSS campaigns were carried out in the established geodetic network between the years of 2017 and 2020. While the first four of the GNSS campaigns were carried out in 2017, the other four were achieved in 2018, 2019, and 2020. In addition, except for the GNSS sites located on the roof of the hospital building, they were destroyed as a result of landscaping due to deformation. For this reason, observations could not be collected at these stations in 2020 and these consist of a total of seven periods of observations. Each campaign was conducted by collecting at least 8 h of observations on two consecutive days. The Receiver Independence Exchange (RINEX) observation files obtained from campaigns were processed with the GAMIT/GLOBK V10.71 academic analysis software [30]. The analyses were carried out in three basic steps. In the first step, the relative coordinates were estimated based on the weighted least squares algorithm using the ionosphere-free linear combination (LC) of the phase observable by the GAMIT module. The orbital and clock parameters were obtained from the International GNSS Service (IGS), and the minimum constraint procedure was used for ambiguity fixing in 5 cm for both horizontal and vertical directions. In the second step, reference frame definition was performed for the daily solutions by using the GLRED module. Then, a seven-parameter Helmert transformation was applied, and its parameters were estimated by means of 10 IGS stations located about a thousand km around the study area with coordinates and the velocity defined in ITRF08. Thus, the reference frame definition was performed, and the coordinate time series were created. In the last step, the daily solutions were combined using the Global Kalman filter (GLOBK) module to estimate the co-seismic deformation. Thus, the GNSS site coordinates and velocities from a combined solution comprising the daily loosely constrained estimates, EOP values, orbit data, and their covariance was estimated. Both coordinate time series and Kalman filter results were normed by assuming the GUMU station, located 7 km away from the deformation area, as fixed in order to eliminate tectonic effects. In order to ensure that the deformation behavior is similar at all stations and to avoid time series confusion, the time series of station NKT1 is shown in Figure 11 as an example. The Kalman filter results are given numerically in Table 1 and the deformation vectors with their error ellipses are given in Figure 12.

### 3.3. Drilling and Geophysical Research

**Figure 13:**
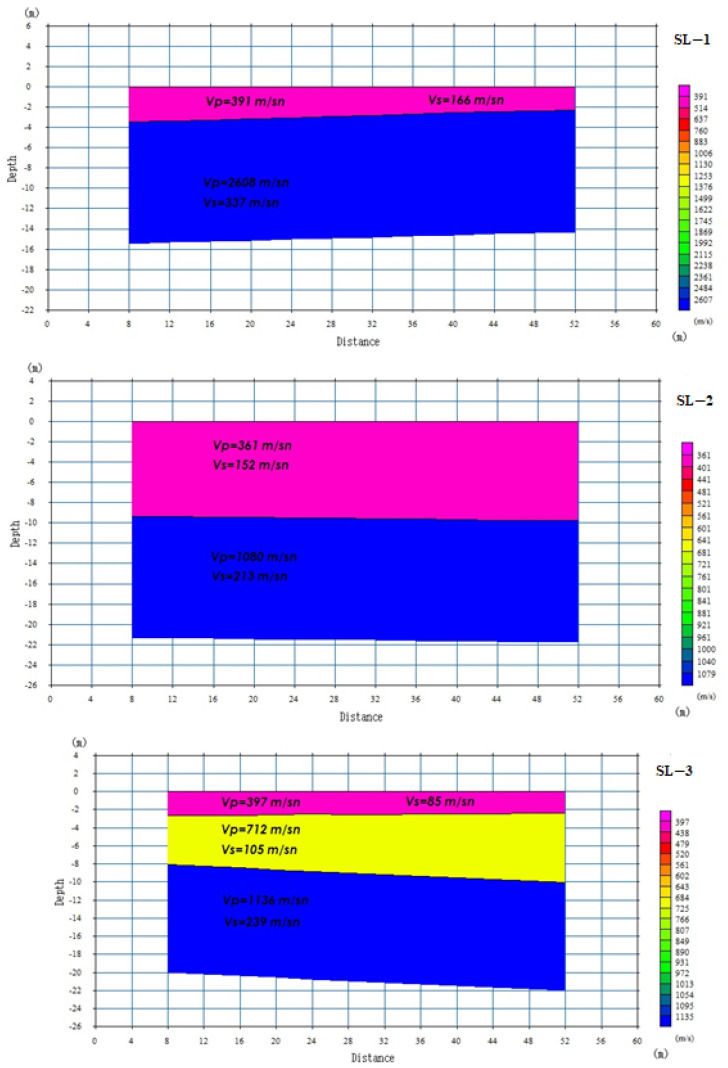
Change sections of seismic fracture lines in the study area with depth.

In order to assess the variation in rock and soil masses within the residential vicinity of the Gümüşhane State Hospital with depth, as well as to determine the dynamic deformation modulus (Edyn) and Poisson’s ratio (νdyn) of the rock masses, a total of six research boreholes and three lines were utilized by employing the seismic refraction (refer to Figure 13) and Multichannel Analysis of Surface Waves (MASW) methods.

The seismic refraction was employed to examine underground properties such as changes in lithological units with depth, the presence of underground void structures, and depth to bedrock. In the seismic measurements, a geophone spacing of 4 m was utilized, with an offset distance of 8 m for seismic refraction and 12 m for MASW measurements. Velocity information for P-wave (Vp) and S-wave (Vs) values was obtained from a depth of approximately 30 m. Data collection involved the use of a 24-channel Geometrics seismograph device, 4.5 Hz vertical component receivers, an 8 kg sledgehammer, and a 25 cm radius iron table.

Using the Vp and Vs velocity values from the same layers along the A-A′ line, the dynamic elasticity modulus (Edyn) and dynamic Poisson’s ratio (νdyn) values were calculated using Equations (1) and (2) proposed by [31] (Table 2).

V_dyn = (Vp 2 − 2Vs 2)/2(Vp 2 − Vs 2)(1)

E_dyn = ν (3Vp 2 − 4Vs 2)/(Vp 2 − Vs 2)(2)

The soil parameters obtained for each line in the seismic refraction and MASW measurements made in the study area are given in Table 2.

**Figure 14:**
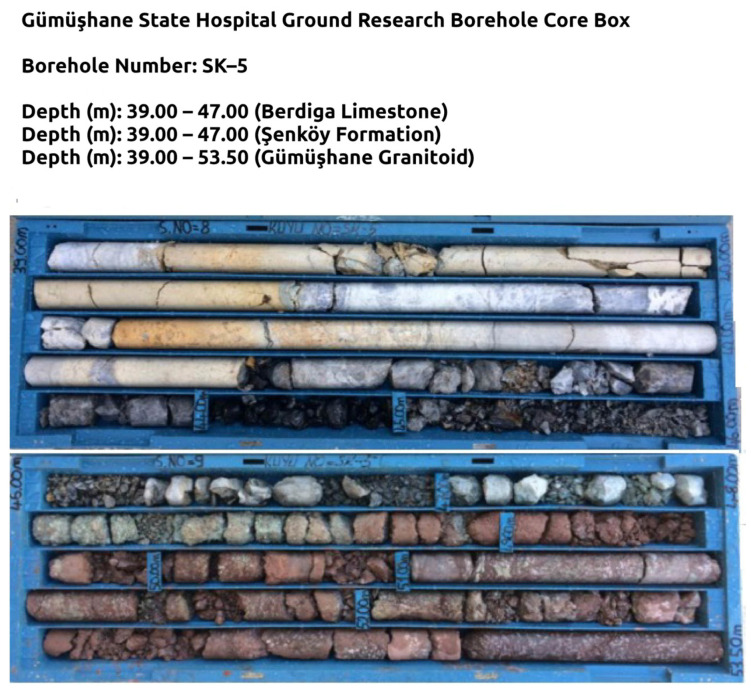
Lithological change in the formation transition observed in drilling well no. BH-5.

Six research boreholes were opened to measure the change of geological units in and around the hospital area with depth, the engineering properties of the rock material, possible shear circle depth, and inclinometer measurements. These research drillings are at depths of BH-1 (15 m), BH-2 (42.5 m), BH-3 (42 m), BH-4 (60 m), BH-5 (70 m), and BH-6 (25 m). In the measurements, a cutting occurred in the clays of the Şenköy Formation at the transition between the Berdiga Formation and Şenköy Formation in drilling wells numbered BH-4 and BH-5. At the depth where this deformation occurs, clay soil belonging to the Şenköy Formation is seen (Figure 14). This shows that the Berdiga limestone mass on which the hospital building sits was exposed to horizontal movement on the clay soil of the Şenköy Formation, thanks to the additional load created by the building construction on the foundation ground.

## 4. Evaluation of Slope Stability

In order to assess the potential risks posed by cracks in the hospital building, the parking lot, and surrounding landscape areas, as well as ground deformations on the slopes, thorough examinations were conducted on the slopes beneath the hospital. Research pits, drillings, and geophysical tests were conducted to determine the properties of the rock mass and ground. Physico-mechanical and elastic parameters (refer to Table 3) were derived through laboratory tests conducted on samples collected from field surveys, geophysical measurements, and drillings.

In the analysis, ASTM (2005) test standards were taken into account to determine the consistency limits of clays belonging to the Şenköy Formation, and ASTM (2011) test standards were taken into account in the shear box test to determine the cohesion and friction angle. The geological strength index (GSI) value, which is widely used to determine the mechanical properties and mass constants of the rock mass in the finite element method, was determined using the numerical GSI graph proposed by Hoek et al. The charts and equations recommended by ASTM and Hoek et al. [32,33,34,35] were used to determine the rock mass constants (mb, s, a), GSI value, rock material constant (mi) and disturbance factor (D).A horizontal ground acceleration of 0.2 g, as determined for the study area from the Turkey Earthquake Map prepared by the General Directorate of Disaster Affairs [36], was employed in the analyses.

The study utilized both the limit equilibrium method (LEM) and the finite element method (FEM), which are widely employed for slope stability analysis. Section A-A′, identified as the most critical segment for the slope stability assessment, was evaluated using computer programs Slide2 and RS2 [37]. In the limit equilibrium (LE) method, the Bishop [38], GLE–Morgenstern–Price [39], Spencer [40], and Janbu [41] methods were employed. For the numerical analysis method, the finite element-based shear strength reduction (FE-SSR) method was applied using the RS2 computer program. Both methods evaluated conditions before the construction of the hospital in the study area and after construction under the influence of seismic loading [42].

**Figure 15:**
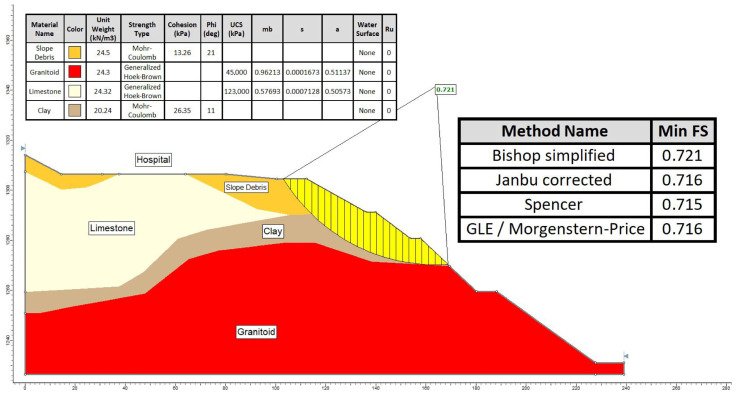
Limit equilibrium analysis in section A-A′ before construction.

**Figure 16:**
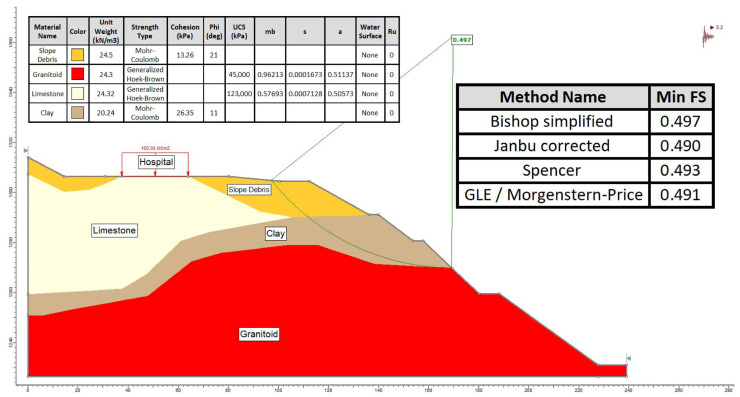
Limit equilibrium analysis in section A-A′ after construction (under the influence of building load + seismic load).

The analyses focused on Section A-A′, the most critical segment, evaluating slope stability before hospital construction (Figure 15) and conducting limit equilibrium analyses for conditions after construction under the influence of seismic loading (Figure 16).

Given that the safety factors (FSs) were found to be less than 1 under the influence of seismic loading in the slope, as determined using limit equilibrium analysis methods both before and after the construction of the hospital, it was concluded that instability was evident in the hospital vicinity and slopes in both scenarios.

**Figure 17:**
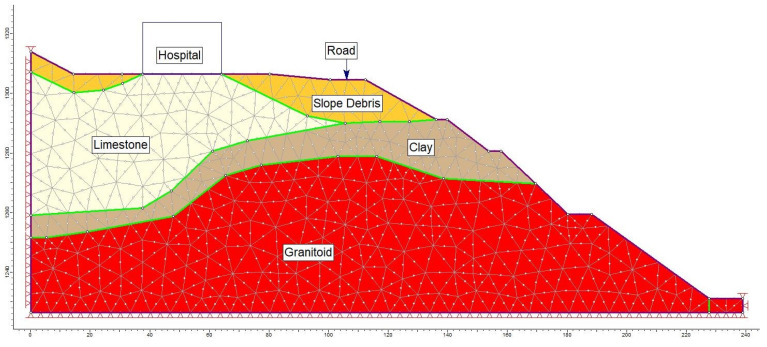
Change in A-A′ section line with depth.

**Figure 18:**
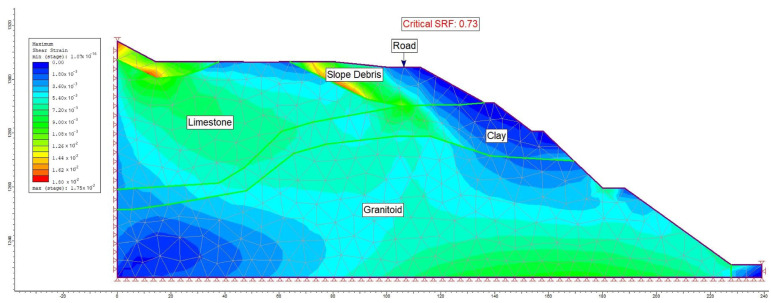
Maximum shear stress analysis occurring in section A-A′.

**Figure 19:**
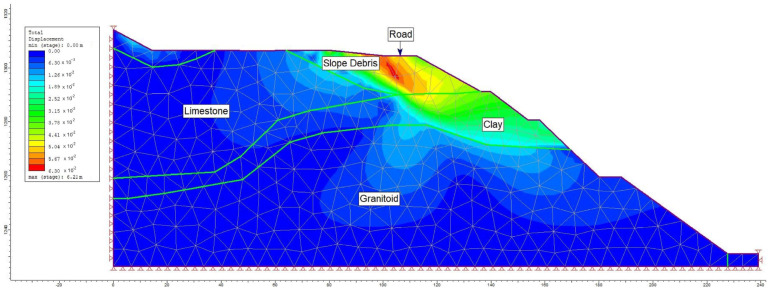
Total displacement analysis in section A-A′.

Moreover, alongside the limit equilibrium analysis method, a numerical analysis approach was employed to assess the failure mechanism within the study area. A graduated triangular network type with six nodes was adopted for the A-A′ section line (refer to Figure 17). Within this model section, analyses were conducted for maximum shear stress (refer to Figure 18) and total displacement (refer to Figure 19) under pre-construction conditions. The numerical analysis revealed a safety factor (SRF) of 0.73 for the evaluated slope, with the total displacement ranging between 3 cm and 6 cm.

**Figure 20:**
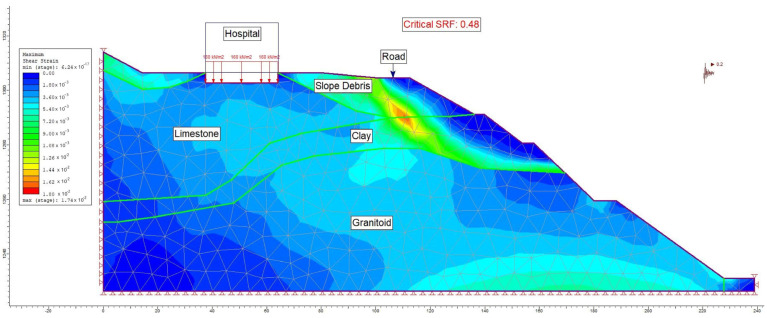
Maximum shear stress analysis occurring in section A-A′ after construction (under the influence of seismic load).

**Figure 21:**
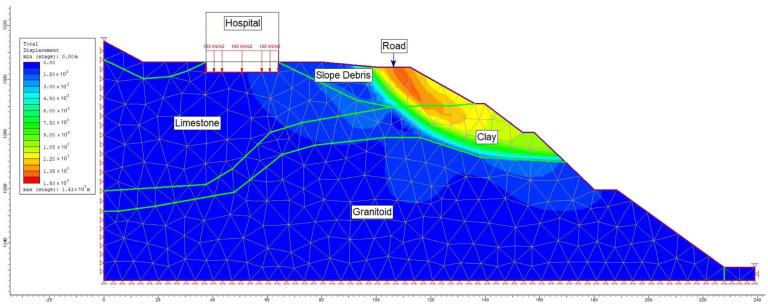
Total displacement analysis in section A-A′ after construction (under the influence of seismic load).

For the post-construction seismic load scenario in section A-A′, analyses were conducted for maximum shear stress (refer to Figure 20) and total displacement (refer to Figure 21). Following the numerical analysis, the safety factor (SRF) for the evaluated slope was determined to be 0.48, with the total displacement ranging between 6 cm and 15 cm.

**Figure 22:**
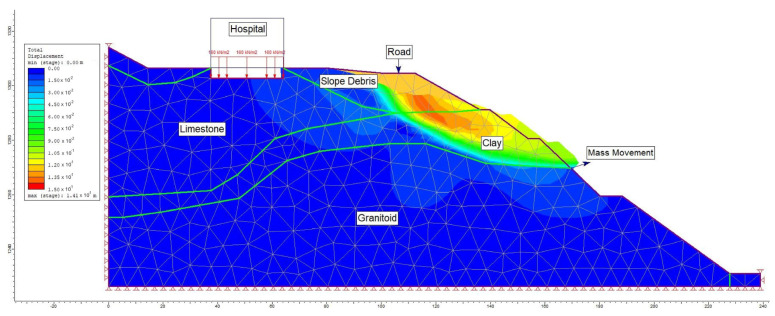
Total displacement simulation in section A-A′ after construction (under the influence of seismic load).

Furthermore, in the deformation analysis, the simulation of the sliding mass indicated that the sliding depth occurred within the clays of the Şenköy Formation and at the border of the Gümüşhane Granitoid (refer to Figure 22). This finding was further corroborated by field investigations.

## 5. Discussion

A detailed examination was conducted on the deformations observed in the hospital building and its surrounding areas, situated within the old landslide zone, along with the stress cracks that developed over time, elucidating the underlying failure mechanism. To this end, a comprehensive array of methodologies including drilling studies, laboratory experiments, geophysical measurements, geodetic monitoring with a GNSS, and inclinometer measurements were employed in the study area.

Upon evaluating the results of drilling operations and inclinometer measurements conducted in response to the observed cracks in the hospital building and landscape openings (refer to Figure 6 and Figure 7), it was discerned that ground deformations existed within the Berdiga limestones and at the transition to the Şenköy Formation (refer to Figure 8 and Figure 9). Inclinometer measurements further revealed the presence of multiple slip circles within the moving mass, while ground cuts were observed along the boundary of the Berdiga limestones and the Şenköy Formation.

The GNSS results indicated horizontal deformations with an annual rate of up to 3 cm at Gümüşhane State Hospital. Vertical movements, as indicated in Table 1, were found to be insignificant, not surpassing their sigma values, whereas a horizontal movement predominantly in the N-E direction was noted (Figure 12). The time series data depicted in Figure 11 revealed a linear pattern of deformation over time. The Kalman filter results presented in Table 1 highlight a distinct direction and magnitude of deformation at the NKT6 station in Section 2 compared to others. Notably, during on-site inspections, significant gaps were observed in this particular section of the structure between Section 1 and Section 2 (Figure 6a,b).

Upon evaluating the failure mechanism within the mass exhibiting deformations with a low shear rate in the study area, through both limit equilibrium and numerical analysis methods, it was ascertained that the safety factor was <1 in both approaches, indicating instability within the examined A-A′ slope section. In the limit equilibrium analyses performed on the A-A′ section line, it is seen that the slope failure occurred on the slopes and the slip circle did not include the hospital building. However, the instability assessment made using the finite element method on the same section line determined that the deformations reached the boundaries of the hospital building. Inclinometer and GNSS observations played a major role in eliminating this uncertainty. In the deformation analysis conducted on the critical A-A′ section, it was observed that the failure extended beyond the slope, reaching the perimeter of the hospital, with the simulated mass movement aligning precisely with the shear zone identified by inclinometer measurements (Figure 22). Considering the stability analyses, total displacements along the critical section line identified within the study area, as well as aboveground and underground monitoring, field observations, and the coherence of obtained horizontal displacements (inclinometer and GNSS), it was concluded that the hospital, constructed within the fossil landslide area, triggered the reactivation of the mass movement.

## 6. Conclusions and Recommendations

Following geological investigations in the study area, it was determined that the site is a relic landslide area from previous years. The construction of the hospital within this zone imposed additional weight on the mass, leading to renewed movement at a slow sliding pace within the stable landslide mass, influenced by surface and groundwater dynamics. Through inclinometer measurements and geodetic monitoring, the velocity and direction of cracks in the hospital building and surrounding deformations were assessed. It was found that the surface slip occurred predominantly in the N-E direction at a rate of approximately 3 cm, consistent with inclinometer readings. The examination of stability using four distinct limit equilibrium analyses and numerical methods revealed a displacement ranging from 3 cm to 6 cm prior to hospital construction, while the post-construction analysis under seismic influence showed displacements ranging from 6 cm to −15 cm. The consistent safety factor < 1 across all analyses suggests the reactivation of the fossil landslide area.

In this study, it has been demonstrated that evaluating engineering structures to be built in such areas with different stability analysis methods before construction will yield more accurate results. While it was observed in the limit equilibrium analyses that the instability that would only occur on the slopes, in the results of the numerical analysis performed on the same section line, it was determined that the slip circle progressed to the foundation of the hospital building. This situation was also confirmed by the cut that occurred in the inclinometer well that was opened in front of the hospital.

One crucial measure for landslide risk mitigation involves minimizing water infiltration into the ground. Surface water should be diverted away from the study area by constructing drainage ditches, particularly behind the hospital building. No new excavations or constructions increasing potential loads should be permitted beneath or above the hospital. To mitigate sliding velocity, rubble on the slope behind the hospital must be cleared, and preventative measures such as diaphragm walls or gradual bored pile applications should be considered to halt sliding movements at an approximately 30 m depth, below the identified slip circle depth determined by inclinometer readings (at the border of the Şenköy Formation and Berdiga limestone). Using the hospital in this way without taking these precautions will cause possible loss of life and property.

By integrating inclinometer readings and GNSS observation, geological investigations, and advanced stability analyses, we provide valuable insights applicable to similar geological and engineering contexts globally. The findings underscore the importance of thorough pre-construction assessments in landslide-prone areas to mitigate risks effectively. This approach not only enhances the understanding of landslide dynamics but also informs tailored strategies for infrastructure resilience in diverse geological settings. Thus, our study contributes essential knowledge for improving practices in geological hazard management and urban planning, promoting safer and more sustainable development in vulnerable regions worldwide.

## Figures and Tables

**Table 1:** Summary velocity estimation with the sigma values from GLOBK module (in mm/year).

Station	North	σ (mm)	East	σ (mm)	Up	σ (mm)
NKT1	14.96	1.19	23.75	1.38	5.06	5.64
NKT2	17.02	1.12	20.75	1.32	1.95	5.29
NKT3	17.19	0.87	26.03	1.03	−5.64	3.78
NKT4	16.04	0.83	23.34	0.99	8.42	3.58
NKT5	15.06	1.06	25.65	1.29	−8.72	4.91
NKT6	9.89	0.85	3.72	1.03	0.58	3.75
NKT7	13.20	1.23	22.50	1.45	−7.18	6.07
KORK	17.72	1.91	28.18	1.93	−4.64	5.28
ISTI	20.70	1.26	30.53	1.52	−7.68	5.62
KAYA	21.71	2.00	35.32	2.28	−10.21	9.02
ARKA	13.20	2.24	22.50	2.36	−7.18	6.07
GUMU *	0.00	0.00	0.00	0.00	0.00	0.00

* GUMU was used as a fixed station to generate deformation values and velocities.

**Table 2:** Vp and Vs velocities and dynamic elastic parameters of the A-A′ section.

Seismic Refraction Line	Layer Number	Depth (m)	Vp (m/sn)	Vs (m/sn)	E_dyn_ (kg/cm^2^)	Poisson Ratio (n_dyn_)
SL-1	1	4	391	166	1054	0.39
	2	16	2608	337	7495	0.49
SL-2	1	10	361	152	868	0.39
	2	22	1080	213	2383	0.48
SL-3	1	3	397	85	295	0.48
	2	8	712	105	525	0.49
	3	21	1136	239	3032	0.48

**Table 3:** Parameters used in numerical analysis and limit equilibrium analysis.

Lithology	GSI	E_i_ (GPa)	σ_ci_ (MPa)	mi	γ kN/m^3^	ν	D	E_m_ (MPa)		Hoek–Brown Constants		
										mb	s	a
Granitoid	40	14.93	45	26	24.3	0.24	0.7	869.38		0.962	0.00017	0.511
Limestone	47	23.13	123	8	24.32	0.36	0.7	2050.78		0.577	0.0007	0.506
Lithology	γ (kN/m^3^)			Elasticity Modulus, E_m_ (MPa)		Poisson’s Ratio, ν		c (kPa)	(ϕ°)	Horizontal Seismic Load (g)		
Slope Debris	24.50			74.80		0.49		13.26	21	0.2		
Clay	20.24			57.00		0.48		26.35	11	0.2		

## Data Availability

Data are contained within the article.
